# Carnivory during Ontogeny of the *Plagioscion squamosissimus*: A Successful Non-Native Fish in a Lentic Environment of the Upper Paraná River Basin

**DOI:** 10.1371/journal.pone.0141651

**Published:** 2015-11-02

**Authors:** Mayara Pereira Neves, Rosilene Luciana Delariva, Ana Tereza Bittencourt Guimarães, Paulo Vanderlei Sanches

**Affiliations:** 1 Programa de Pós-Graduação em Conservação e Manejo de Recursos Naturais, Centro de Ciências Biológicas e da Saúde, Universidade Estadual do Oeste do Paraná, Cascavel, Paraná, Brazil; 2 Pós-Graduação em Recursos Pesqueiros e Engenharia de Pesca, Universidade Estadual do Oeste do Paraná, Toledo, Paraná, Brazil; Bournemouth University, UNITED KINGDOM

## Abstract

This study evaluated feeding patterns and ontogenetic variations in a non-native fish species (*Plagioscion squamosissimus*) in an isolated lake in the Upper Paraná River floodplain. Quarterly samplings were performed from April 2005 to February 2006 using plankton nets to capture larvae, seining nets for juveniles, and gill nets and trammel for adults. Stomach contents (n = 378) were examined according to the volumetric method in which the volume of each food item was estimated using graduated test tubes or a glass counting plate. During early development (larval stage), *P*. *squamosissimus* consumed mainly Cladocera and Copepoda. Juveniles showed a more diverse diet, including shrimp (*Macrobrachium amazonicum*), fish, aquatic insects (Trichoptera, Ephemeroptera, Chironomidae and pupae of Diptera) and plants. It was notable the high proportion of cannibalism (23.3%) in this stage. Adults consumed predominantly shrimp and fish. The use of food resources varied significantly between development stages (ANOSIM; r = 0.458; *p*<0.005), showing changes in food preferences during ontogeny. The Similarity Percentage Analysis (SIMPER) indicated that Cladocera and Copepoda were responsible for the differences observed between the larval stages of pre-flexion, flexion and post-flexion. *M*. *amazonicum* and Chironomidae were responsible for the differences between juvenile and larval stages, while *M*. *amazonicum* and other fishes caused the differences between adults and other ontogenetic stages. These results are confirmed by the relationship between standard length and developmental periods (ANCOVA; r^2^ = 0.94; *p*<0.0001). In general, there were low values of trophic niche breadth. The essentially carnivorous habit from the early stages of *P*. *squamosissimus* and the predominant use of *M*. *amazonicum* by adults have important roles in feeding patterns of the species, suggesting a major contribution to its success and establishment, especially in lentic environments.

## Introduction

*Plagioscion squamosissimus*, Heckel, 1840, (Sciaenidae, Perciformes), commonly known as curvina, is considered carnivorous in its natural habitat in the Amazon basin. Initially, this species was introduced into northeastern Brazil, and was subsequently released by the Energy Company of São Paulo (CESP) in 1967 in the Pardo River where it spread throughout the Paraná River basin [1, 2, 3]. In all environments, it has become successful, being among the dominant species in various reservoirs, where, in some, it is also one of the main species for commercial fishing [4, 5, 6, 7].

The significance of this species in the Upper Paraná River, given by its high abundance in regions of the Itaipu Reservoir [8, 9] and in several environments of the floodplain upstream, including environmental protection areas like the Parque Nacional de Ilha Grande [10, 11] and the Parque Estadual das Várzeas do Rio Ivinhema [12], places this species in a prominent position, especially because of its economic importance and ecological interactions. In this context, it becomes essential to address its biological characteristics, especially its feeding habits in all stages of the life cycle.

The initial ontogeny of fish can be considered a series of vulnerable periods; the most important is the transition between endogenous and exogenous feeding, which is a period where the survival of the young depends on the quantity and availability of adequate food at the first feeding. Therefore, the success or failure of a population is largely determined during the early life history [13]. Therefore, information on the diet of a species in the larval period, as well as adults, enables the elucidation of its ecological relationships with other species. In addition, assessing the trophic position along the ontogenetic development of non-native species allows researchers to find evidence relative to their success and establishment in environments, as well as the impact of their presence on other species. Moreover, studies on the biology of non-native species in protected areas became relevant, mainly when considering biological invasions with consequent fauna homogenization as one of the main threats and causes of biodiversity loss [14, 15].

The majority of studies on *P*.*squamossimus* have reported the trophic ecology of adults in reservoirs [7, 16, 17, 18], but information on feeding during initial development is lacking, primarily considering natural environments. Moreover, even more incipient studies have concurrently evaluated the diet of larvae, juveniles and adults in the same space and time. Thus, evaluating the feeding during ontogeny allows researchers to assess the way growth and morphological changes interfere with trophic relationships, and to understand the interactions in a particular community [19, 20, 21, 22].

The selectivity and the ability to effectively capture food are important factors that contribute to the success and establishment of a species [20, 23, 24]. Based on this premise, this study tested the hypothesis that *P*. *squamosissimus* consumes different food resources throughout its life cycle according to body size. In this context, we analyzed the diet of this species in an isolated oxbow lake of the Parana River. It is, therefore, essential to understand the ontogeny influences on the success of the species in such a biotope. Specifically, the objectives of this study were: (i) to describe the diet of *P*. *squamosissimus*at different stages of its life cycle (larval to adult); (ii) to check for differences in diet according to ontogeny; (iii) to determine the food resources that contributed most to the diet in each stage of the life cycle; (iv) to show the relationship between the length (Ls) at different stages of the life cycle and the consumption of different food resources; and (iv) to evaluate changes in ecological niche breadth according to ontogeny.

## Material and Methods

### Ethics statement

Fish samples were collected with authorization from Instituto Brasileiro do Meio Ambiente e dos Recursos Naturais Renováveis (IBAMA) (process number 02001004095/04-98, license numbers 120/2004 and 204/2005), under the responsibility of the researcher Rosilene Luciana Delariva. This study was approved by Ethics Committee on Animal Use of the Universidade Paranaense (CEPEEA) (Permit Number: 13/04) and was conducted in accordance with protocols in their ethical and methodological aspects, for the use of fish.

### Study area

**Fig 1:**
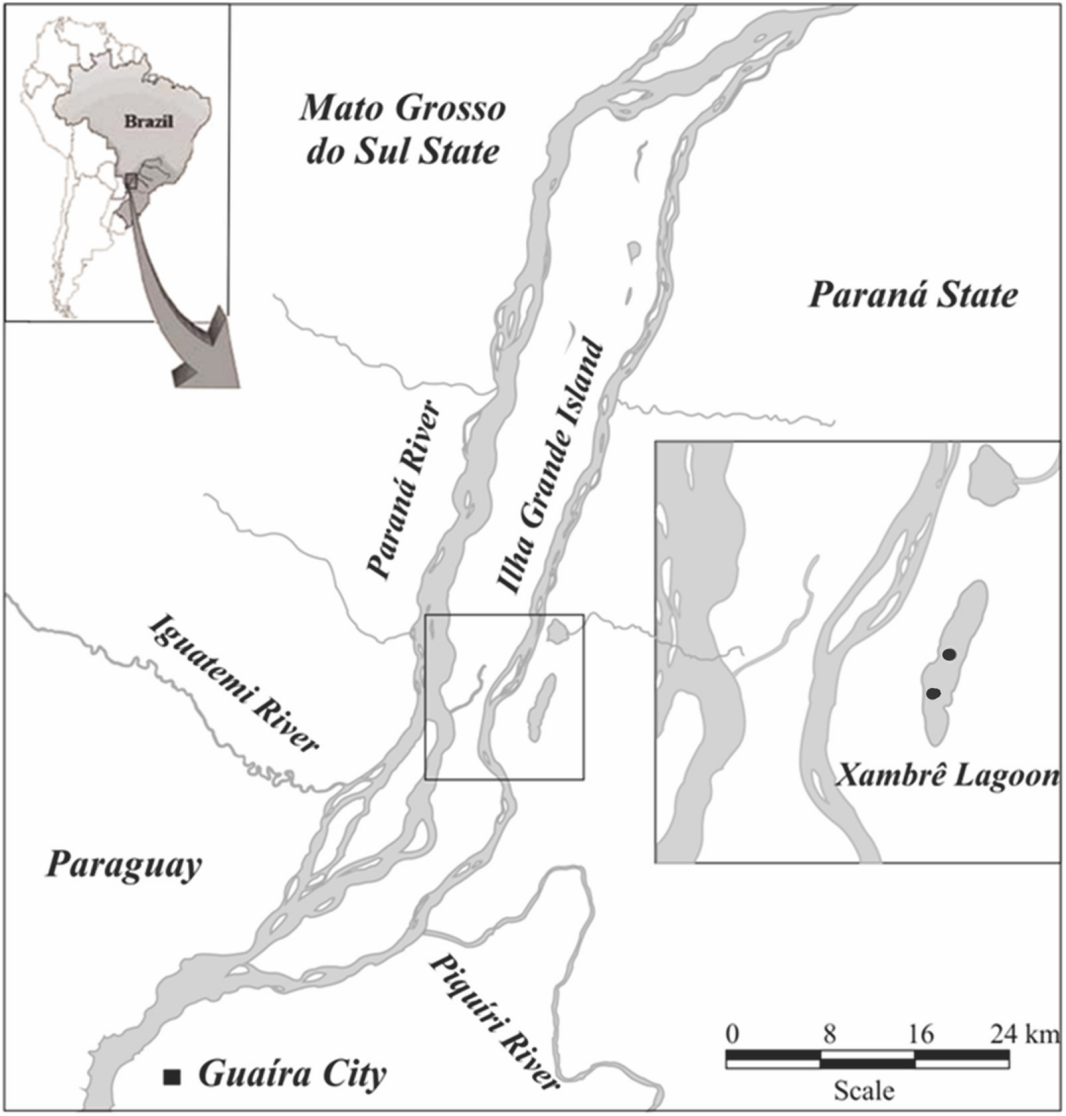
Study area: Upper Paraná River floodplain–Xambrê Lake, Parque Nacional de Ilha Grande, Paraná State, Brazil.

The Xambrê Lake is located on the left bank of the Paraná River, within the limits of the Parque Nacional de Ilha Grande (Fig 1). The National Park is formed by a fluvial archipelago with hundreds of islands. The alluvial deposits that originated in the floodplain of the Upper Paraná River are related to the first wet event of the Superior Quaternary, while the current lagoons and oxbow lakes in of the National Park area were formed more recently in the second wet event (1500 AP)[25]. The studied environment is an isolated oxbow lake, approximately 5 km long, 1 km wide and about 3 meters deep. It has an extensive floodplain area on its right margin that separates it from the Paraná River. Currently, it is maintained by groundwater and a small stream. Its connection with the Paraná River was sporadic in years with very high floods; the last record was in 1996. However, after the construction of the Porto Primavera Reservoir, there were no such floods to connect the lake with the Paraná River [10, 26]). Its right bank is colonized by shrubby vegetation and aquatic macrophytes, while the left bank is bordered by arboreal vegetation.

### Sampling

Fish (larval, juvenile and adult developmental stages) were collected quarterly from April 2005 to February 2006, at two sites in the Xambrê Lake. Samples of the larval stages were taken at night with a plankton net (0.5 mm mesh size). The net was towed on the water surface behind a boat at low speed for 10 min in the subsurface waters (approximately 20 cm deep). Samples were transferred to polyethylene flasks and preserved in 4%formalin buffered with calcium carbonate. Juveniles were collected using the seine operated in the left marginal area of the lake, during dusk (19.00h). Adult specimens were caught using gill nets (mesh sizes of 2.4, 3, 4, 5, 6, 7, 8, 9, 10, 12, 14, and 16 cm between opposite knots) and trammel nets (with inner mesh sizes of 6, 7, and 8 cm between opposite knots), both 20 m long. The effort was the same at each site. Nets remained set for 24 h and were checked every 8 h (morning, afternoon, and night).

### Laboratory procedures

Identification of fish larvae was performed according identification keys [27], and individuals were characterized according to the degree of flexion of the terminal region of the notochord and development of the fins and separated into stages of pre-flexion, flexion, and post-flexion [27, 28].We measured the standard length (SL) of fish larvae specimens with an ocular micrometer under a stereomicroscope. Adult and juvenile specimens were identified [29], weighed on analytical balance, measured (standard and total length, in cm), and then eviscerated. Voucher specimens were deposited in the fish collection of Gerpel (Grupo de Pesquisas em Recursos Pesqueiros e Limnologia), Universidade Estadual do Oeste do Paraná, Brazil (lot Cig 2380).

### Diet composition analysis

To characterize the diet of larval stages, the digestive tube was removed through a longitudinal cut in the abdomen using a probe or scalpel, when necessary. Digestive tubes were then opened on blade and covered with coverslip. Posteriorly, fields were randomly chosen, and the items were identified, counted, summed and multiplied by an approximate value of a known volume, obtaining the total volume of items in each digestive tube analyzed. For individuals at pre-flexion and flexion stages all the digestive tube content was analyzed. Larvae at the post-flexion stage that already showed a clear differentiation of the digestive tube, had only the stomach content and 2/3 of the intestine analyzed, due to a high degree of digestion in the final portion.

The diet analysis for juvenile and adult stages was based on the stomach contents of 32 adults and 127 juveniles relating to all stomachs containing food (fullness equal to or greater than 50%). Food items were identified under optical and stereoscopic microscopes to the lowest taxonomic level possible, using the identification keys for algae [30] and invertebrates [31]. In order to quantify the food items, we used the volumetric method; *i*.*e*., the total volume of a food item taken by the fish population is given as a percentage of the total volume of all stomach contents [32], using graduated test tubes and a glass counting plate [33].

### Data analysis

Aiming to evaluate the sampling sufficiency of stomach/intestine contents of *P*. *squamosissimus*, rarefaction curves were constructed for different ontogenetic periods. In this analysis, each point of the curve was the result of a mean value obtained by randomization in the order of the samples, with a standard deviation assigned for each value.

To test the null hypothesis of no difference in diet composition between different developmental stages of *P*.*squamosissimus* summarized by the nMDS (distance matrix), we used an ANOSIM (Analysis of Similarities) based on Bray-Curtis distance. These tests are applied in ecology for comparing the taxonomic composition in more groups of samples [34]. If two stages are different in their use of food resources, we expect smaller distances within each group than between groups.

Finally, we used discriminant analysis (SIMPER-similarity percentage) to determine which food resources were responsible for dissimilarity between groups (developmental stages of the fish life cycle) [35]). We used the Bray-Curtis distances to distinguish the diet composition and participation of each food resource between different stages of development. The general dissimilarity was calculated using all resources, where as the dissimilarity resource-stage was calculated for separated resources.

Food resources and standard length were compared between developmental stages by an Analysis of Covariance, using the volume of food resources as covariate (ANCOVA, *p*<0.05), to reduce the bias of each developmental stage [36].

In order to demonstrate the relative degree of specialization in the diet of the developmental stages, the trophic niche breadth (dietary breadth) was estimated based on the volume of food items, using the standardized Levins’ Index [37], which ranges from 0, when a species consumed only one type of food, to 1, when a species consumed similarly all the food items. This is given by the formula:

$$Bi=[(\Sigma j.P^{2}ij{)}^{-1}-1].(n-1{)}^{-1}$$

where: B*i* = standardized trophic niche breadth; *Pij* = proportion of food item *j* in the diet of species *i*; n = total number of food items.

The rarefaction curves, ANOSIM and SIMPER were run using the software PAST 1.68 [38]. ANCOVA was performed using the software Statistica 7.0 [39].

## Results

### Diet composition

**Fig 2:**
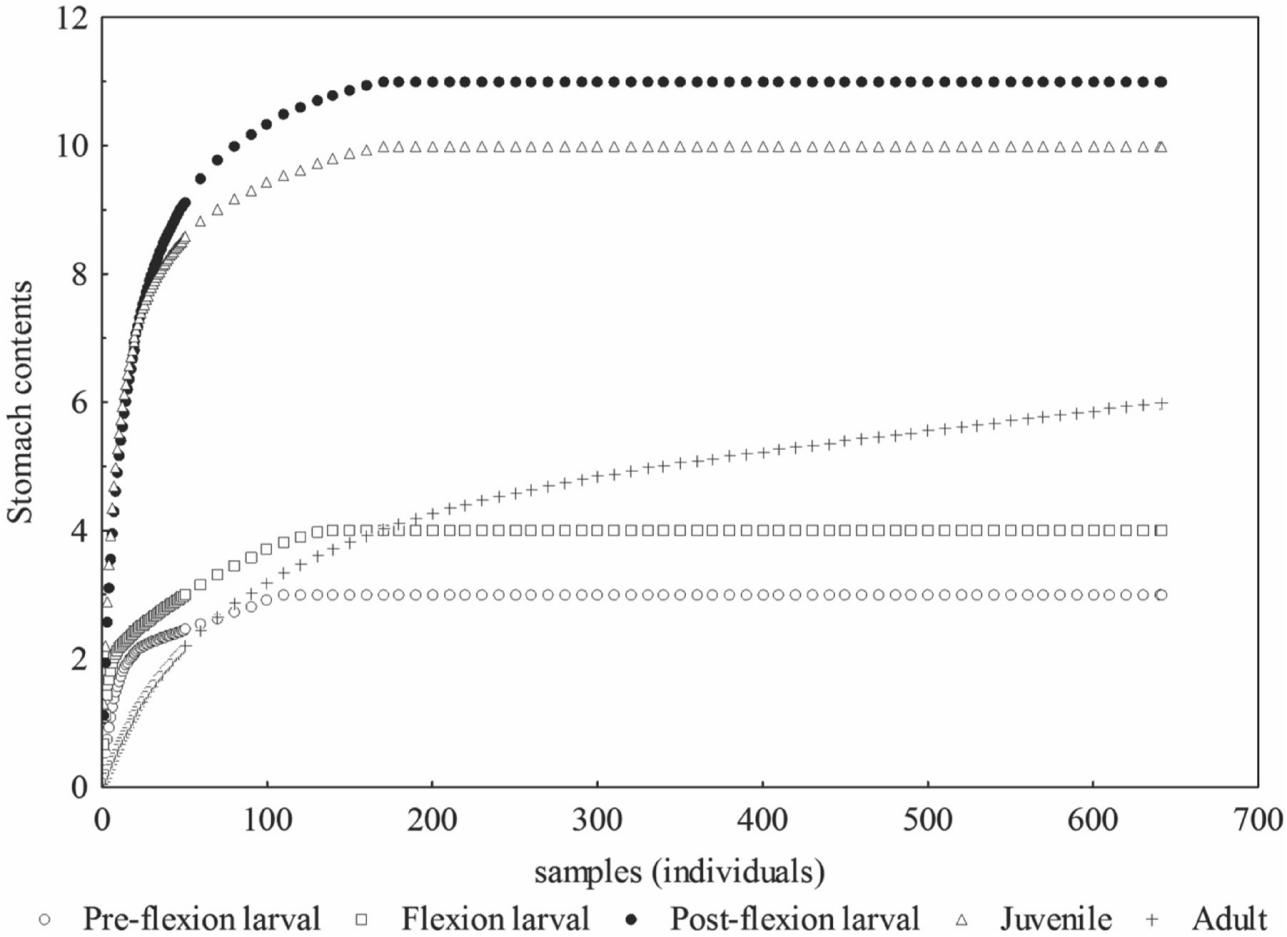
Rarefaction curves according to the number of stomachs with food at different developmental stages of *P*. *squamosissimus* in Xambrê Lake, Upper Paraná River floodplain, Paraná State, Brazil.

The rarefaction curve was asymptotic to each ontogenetic stage sampled, suggesting that the number of individuals analyzed was sufficient to represent the diet of each stage (Fig 2).

The diet of the larval stage was mainly composed of Copepoda (Cyclopoida and Calanoida) and Cladocera; in the pre-flexion stage, the diet also included algae (diatoms) and in the flexion and post-flexion stages, Gastropoda and Chironomidae were also consumed. Juveniles had a more diverse diet, consuming shrimp (*Macrobrachium amazonicum*), fish, aquatic insects (Trichoptera, Ephemeroptera, Chironomidae and pupae of Diptera) and plants. There was considerable cannibalism in the juvenile stage (23.3% consumption of *P*. *squamosissimus* juveniles). Adults predominantly consumed shrimp and fish (Table 1 and S1 Table).

**Table 1:** Food items consumed (percentage volume) by *P*. *squamosissimus* in Xambrê Lake, Upper Paraná River floodplain, Paraná State, Brazil.

Stages	Pre-flexion	Flexion	Post-flexion	Juvenile	Adult
N analyzed stomachs	17	110	92	127	32
Food items					
Bacillariophyta	23.8				
Plant remains				0.3	2.1
Gastropoda		10	7.3		
Copepoda	31	44.5	14.6	0.1	
Cladocera	45.2	44.1	78.1	0.1	
*M*. *amazonicum*				61.6	63.1
Chironomidae		1.4		5.2	
Ephemeroptera				0.9	
Trichoptera				0.1	
Other aquatic insects				2.9	1.4
*P*. *squamosissimus*				23.3	1.6
Other fishes				5.6	31.8

### Ontogenetic variations in the diet

The food resources varied significantly between developmental stages (ANOSIM; Bray-Curtis; 9999 permutations; r = 0.458; *p* = 0.0001). In fact, all distance measures based on the volume of food resources indicated significant differences (*p*<0.05), suggesting that the food preference changes during ontogeny of the species.

The Similarity Percentage (SIMPER) analysis indicated that Cladocera and Copepoda were responsible for the observed differences between the larval stages of pre-flexion vs. flexion vs. post-flexion. *M*. *amazonicum* and Chironomidae accounted for the differences between juveniles and larval stages (pre-flexion, flexion and post-flexion). *M*. *amazonicum* and other fishes were responsible for the differences between adult and other stages of the life cycle (Table 2).

**Table 2:** Values of SIMPER analysis of *P*. *squamosissimus* developmental stages and the most representative food items at each stage.

Pair A x B	Overall average dissimilarity	Most influencial resources (> 50%)	Contribution	Cumulative contribution	Mean volume A	Mean volume B
**Pre-flexion (A) vs. Flexion (B)**	**83.05**	**Copepoda**	**32.45**	**39.07**	**1.12 10–5**	**1.14 10–5**
** **	** **	**Cladocera**	**29.54**	**74.64**	**7.65 10–6**	**1.15 10–5**
**Pre-flexion (A) vs. Post-flexion (B)**	**87.33**	**Cladocera**	**49.72**	**56.94**	**1.12 10–5**	**4.18 10–5**
** **	** **	**Copepoda**	**20.75**	**80.70**	**7.65 10–6**	**7.83 10–6**
**Pre-flexion (A) vs. Juvenile (B)**	**99.89**	**M. amazonicum**	**50.90**	**50.95**	**0.00**	**0.05**
** **	** **	**Chironomidae**	**13.81**	**64.77**	**0.00**	**0.00**
**Pre-flexion (A) vs. Adult (B)**	**100**	**M. amazonicum**	**81.45**	**81.45**	**0.00**	**1.98**
** **	** **	**Other fishes**	**10.36**	**91.81**	**0.00**	**1.00**
**Flexion (A) vs. Post-flexion (B)**	**65.66**	**Cladocera**	**37.71**	**57.42**	**1.14 10–5**	**4.18 10–5**
** **	** **	**Copepoda**	**23.49**	**93.20**	**1.15 10–5**	**7.83 10–6**
**Flexion (A) vs. Juvenile (B)**	**99.85**	**M. amazonicum**	**50.89**	**50.97**	**0.00**	**0.05**
** **	** **	**Chironomidae**	**13.81**	**64.80**	**3.7 10–7**	**0.00**
**Flexion (A) vs. Adult (B)**	**100**	**M. amazonicum**	**81.45**	**81.45**	**0.00**	**1.98**
** **	** **	**Other fishes**	**10.38**	**91.81**	**0.00**	**1.00**
**Post-flexion (A) vs. Juvenile (B)**	**99.77**	**M. amazonicum**	**50.85**	**50.96**	**0.00**	**0.05**
** **	** **	**Chironomidae**	**13.79**	**64.78**	**0.00**	**0.00**
**Post-flexion(A) vs. Adult (B)**	**100**	**M. amazonicum**	**81.45**	**81.45**	**0.00**	**1.98**
** **	** **	**Other fishes**	**10.36**	**91.81**	**0.00**	**1.00**
**Juvenil (A) vs. Adult (B)**	**94.56**	**M. amazonicum**	**74.94**	**79.25**	**0.05**	**1.98**
** **	** **	**Other fishes**	**10.50**	**90.36**	**0.00**	**1.00**

**Fig 3:**
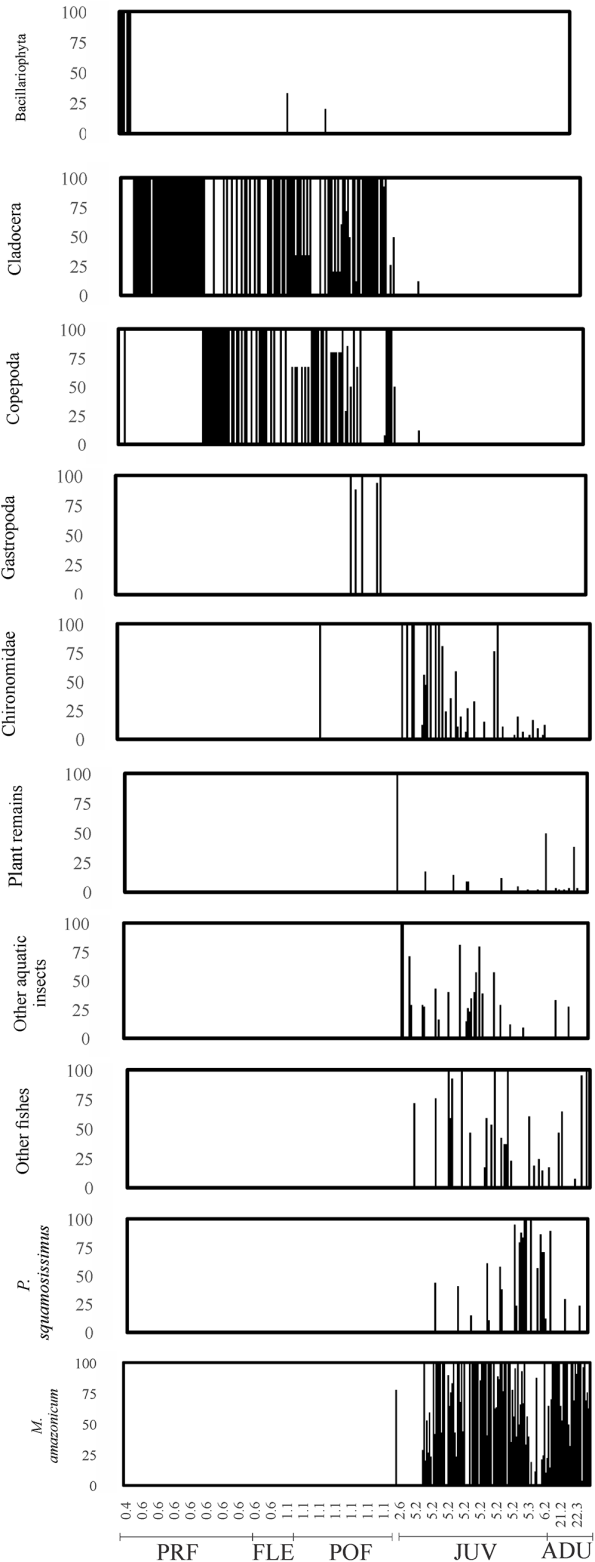
Percentage of the food item in relation to total consumption of food items according to the standard length of *P*. *squamosissimus* in Xambrê Lake, Upper Paraná River floodplain, Paraná State, Brazil.

Values of standard length were significantly different between developmental stages (ANCOVA; r^2^ = 0.94; *p*<0.0001) (Table 3 and Fig 3). Copepoda was a specially adjusted food item to individuals with smaller size (larval stages), while *M*. *amazonicum*, plant remains, Ephemeroptera, Trichoptera and other fishes, including *P*. *squamosissimus*, were adjusted to juveniles and adults.

**Table 3:** Standardized coefficients and confidence intervals (Lower and Upper limit) of ANCOVA and significant values (T and Pr > |t|) for food items, standard length and developmental stages of *P*. *squamosissimus* in Xambrê Lake, Upper Paraná River floodplain, Paraná State, Brazil.

Fonte	Value	Standard error	T	Pr > |t|	Lower limit (95%)	Upper limit (95%)
Copepoda	-0.058	0.013	-4.502	< 0.0001	-0.083	-0.033
Stage—Post-flexion	0.033	0.014	2.293	0.022	0.005	0.062
Trichoptera	0.049	0.013	3.828	0	0.024	0.074
*P*. *squamosissimus*	0.056	0.013	4.25	< 0.0001	0.03	0.082
Plant remains	0.06	0.013	4.58	< 0.0001	0.034	0.086
Ephemeroptera	0.06	0.013	4.712	< 0.0001	0.035	0.085
*M*. *amazonicum*	0.062	0.02	3.189	0.002	0.024	0.101
Other fishes	0.123	0.013	9.209	< 0.0001	0.097	0.149
Stage–Juvenile	0.342	0.015	22.97	< 0.0001	0.313	0.371
Stage–Adult	0.879	0.021	42.04	< 0.0001	0.838	0.92

### Trophic specialization

**Fig 4:**
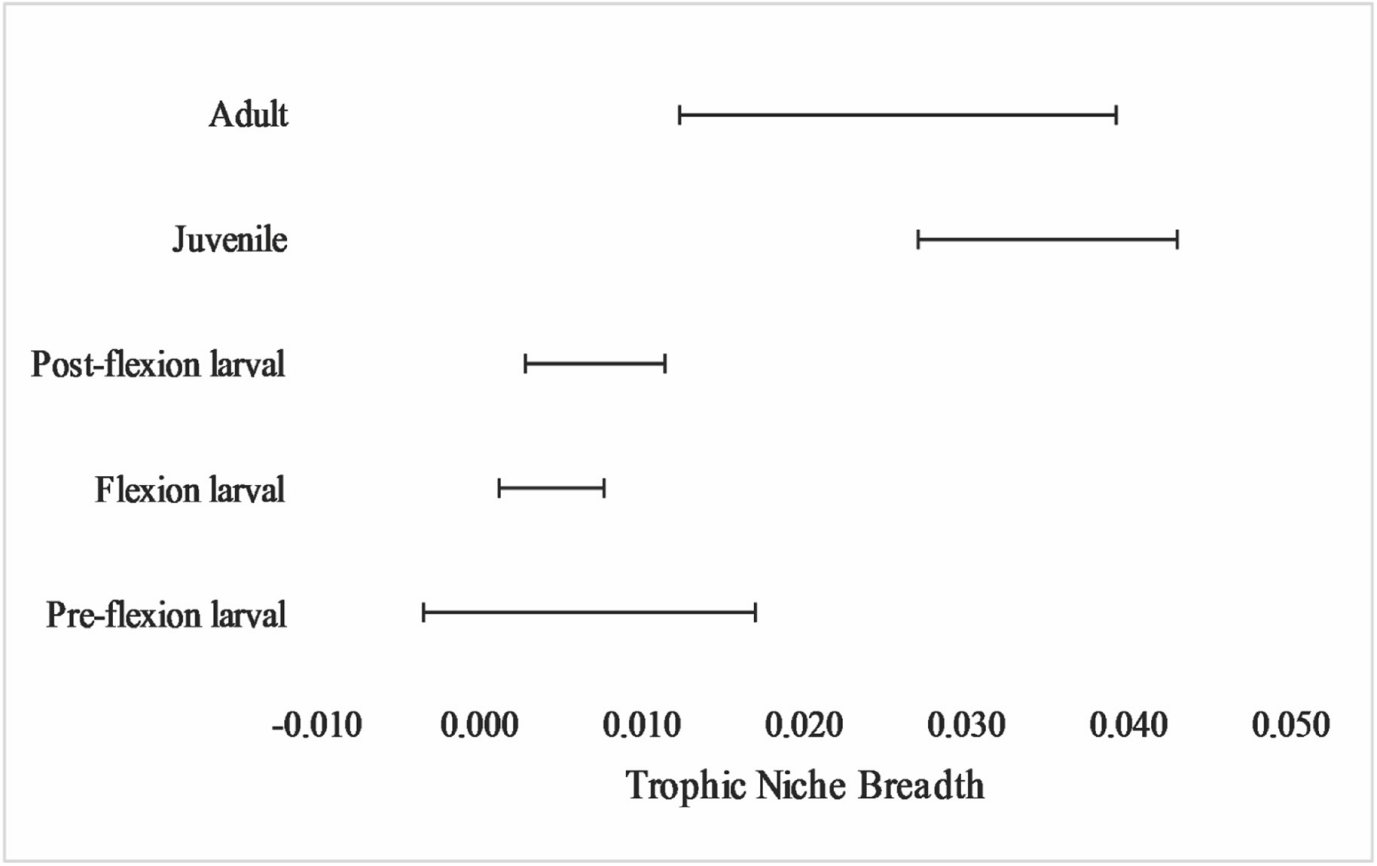
Standardized trophic niche breadth (Levins) for the different developmental stages of *P*. *squamosissimus* in Xambrê Lake, Upper Paraná River floodplain, Paraná State, Brazil.

In general, there were low values of trophic niche breadth, and the early stages (larval stages) showed the lowest values. As the individuals grew, the trophic niche breadth gradually increased with the participation of new food items (Fig 4).

## Discussion

The high consumption of prey was found in all developmental stages. The items recorded in stomach contents were substantially comprised of different taxonomic groups of animals present in the layers of the water column. This result indicates that this species can be considered a generalist carnivorous feeder. In addition, our finding clearly shows that the diet of *P*. *squamosissimus* includes an ontogenetic shift in the selectivity and acquisition of food items, increasing proportions of larger and more evasive prey as fish grow. This widespread observation seems to be a common trend in fish [22, 23], and reinforces the importance of this type of study for understanding not only the trophic requirements in the early stages (most critical),but also factors influencing population sizes of adult fish.

In the early developmental stage (larvae), the species consumed mainly Cladocera and Copepoda at different proportions. These microcrustaceans are large and agile prey, whose capture can be associated with mouth opening size, visual acuity and swimming ability [20, 40]. However, our results showed no difference in diet between larval developmental stages, indicating that, even in the early stages, regardless of reduced swimming ability and visual acuity (especially at pre-flexion) [20], larvae were able to capture these prey. Further, the presence of benthic organisms, such as gastropods, insect larvae and aquatic insects in the stomachs of larvae at flexion and post-flexion stages, as well as of juveniles, indicates that they already have the swimming ability to explore the sediment in search of food. Typically, the most developed stages (especially juveniles) already have well-developed fins, which promote a more effective displacement. After reaching a certain degree of development, larvae migrate to more structured regions such as marginal areas. This migration pattern, known as ontogenetic migration or lateral migration, is described for several fish species [41]). In this type of migration, at least one stage of the life cycle takes place in a different environment from the other. In this way, despite the low swimming ability of the earliest larval stages when compared to adults, the lateral migration during this period would have remarkable trophic consequences. The carnivorous habit, beginning at this stage of the life cycle, is favored in marginal areas that present both shelter and abundant food [20, 21].

The transition between endogenous and exogenous feeding is crucial for the survival of larvae [13, 19], in which there are many morphological changes in the mouth and digestive tract [42, 43]. The carnivorous habit displayed from the earliest developmental stages gives several advantages to the species. This is critical, especially in the larval stage, which is considered the period of the highest vulnerability and mortality [44, 45]. The consumption of energetically advantageous prey allows the larvae to overcome this critical period and reach the juvenile stage successfully [40].

The broader food spectrum of juveniles may be related to greater swimming performance and increased complexity of the buccal apparatus which favors the capture and ingestion of energy-efficient prey, resulting in rapid growth and survival. Besides, *P*. *squamosissimus* has a defined stomach with pyloric caeca in the initial stages [19], which provides the species with the advantage of digesting larger prey. Thus, behavioral and structural adaptations, such as food preference and a complex mouth, are advantageous for capturing mobile organisms, a typical tactic of carnivores [46].

In this study, the high occurrence of cannibalism in the *P*. *squamosissimus* diet is noteworthy. Most studies on the diet of this species did not detect cannibalism, e.g. [3, 7, 16, 21, 47, 48], suggesting that it is not a common feeding strategy. Although cannibalistic behavior has been reported in the literature associated with a wide variety of taxa, habitats and life history strategies, it is particularly well represented in piscivorous [49, 50], where larger individuals predate upon small ones. Thus, the predation by older and unrelated conspecifics accounts for the majority of cases of cannibalism in fish [51, 52]. Herein, however, the ingestion of individuals of their own species was exclusive of juveniles, which may be related to some behaviors commonly displayed by predators: i) taking advantage of the high abundance of juveniles. Even though we do not have data about the availability of prey in the environment, this factor is relevant when analyzing the composition and relative abundance of the fish fauna in marginal areas of the studied lake, where it was observed that *P*. *squamosissimus* was the most abundant species, being essentially represented by immature individuals [26]. In addition, the period of highest cannibalism (stomach contents in late January and March) coincided with the sudden increase in the availability of juveniles, a fact supported by the occurrence of spawning and high densities of larvae of this species in the Upper Paraná River floodplain, especially during the high water-level season associated with high temperatures [10, 53, 54,55]; ii)the intake of prey being advantageous for growth and development, thus maintaining the energy within the population. In this aspect, conspecifics represent a diet of high nutritional quality, with minerals and amino acids in the optimal proportion for maximal growth [51, 56]. As a result, more importantly, cannibalism at an early age accumulates energy for growth, so the species can escape the predation window of other predators; iii) the regulation of population abundance, which allows the species to minimize the intraspecific competition in the adult stage. The regulatory role of cannibalism in experimental populations has been clearly demonstrated [57], but the situation in wild populations is still largely controversial [49, 51]. In this sense, although it is hard to observe the latter two behaviors in natural populations, in the case studied here, none of them can be discarded. Additionally, the typed isolated oxbow lake can also be another factor that induces the cannibalisminfluenced by the higher population density of juveniles because of the lack of chances for dispersal. Furthermore, possibly all the factors above must act concurrently.

For the diet of both juveniles as adults, *M*. *amazonicum* was an important resource, as verified in other studies [7, 16, 21, 47, 48]. This food preference is supported by the high participation of shrimp in the diet of this species in its original environment (Amazon basin) [58]. In addition to the preference, this important contribution may be related to the high abundance of this resource in the studied lake [10, 59]. Along with these factors, the body morphology of curvina, likewise, can assist and facilitate the capture of this prey [21, 60]. The presence of an extensible terminal mouth allows for capture in structured places, especially in shallow environments colonized by aquatic macrophytes, as observed in the sampling sites and reported in another study in the Upper Paraná River floodplain, upstream of the current study area [47].

In the adult stage, although shrimp consumption remained above 50%, the consumption of fish increased. This can be explained by the optimal foraging theory, which predicts that the predator tends to preferentially consume types or sizes of prey that are energetically more favorable as to maximize its fitness [23, 61]. In general, predators select small prey when they are at greater abundance and availability. To compensate for the energy cost, the number of small prey should be substantially greater than the number of larger prey [21].

Accordingly, the characterization of feeding habits during the ontogeny of *P*. *squamosissimus* provides information that associates the food items with the stages of the life cycle, showing food preferences throughout the ontogenetic development. The diet included both increased prey size and changes in types of prey related to growth and development, consistently demonstrated by the model (ANCOVA). The obtained results clearly show the direct relationships between developmental stages and the type of items consumed. It was possible to verify food preferences associated with body characteristics at each stage of the ontogenetic development.

The carnivorous behavior from the early stages is supported by low values of trophic niche breadth. Meanwhile, along the ontogenetic development, food items are replaced and added, with greater variation in juveniles because of the greater richness of the items consumed. The feeding diversity in juvenile fish is commonly higher than during the larval period, and there is often an increased importance of species-specific dietary traits. At this stage, the acquisition of energy for growth and survival is extremely important. However, some limitations on morphological traits, such as mouth size and habitat where juveniles occur (marginal areas), lead to the ingestion of several smaller items, like shrimp and aquatic insects. Furthermore, adults are more selective in capturing prey, restricting their diets to foods with a favorable energy balance, since their morphological characteristics reflected in swimming ability and body size enable the capture of larger, more agile prey, such as fish.

In conclusion, this study provided novel insights into the feeding ecology of curvina. The results support the initial hypothesis that *P*. *squamosissimus* consumes different food resources according to body size, directly related to the degree of development, but exhibiting carnivorous habits from the early stages. Moreover, the predominant consumption of *M*. *amazonicum*, by juveniles and adults, as well as the morphological and behavioral characteristics associated with the use of energetically favorable food items (including significant cannibalism among juveniles), should play a key role in the colonization of this species, especially in lentic environments. The successful colonization of non-native species is expected, as is the case with curvina, mainly in situations where the absence of predators, the abundance of preferential food resources and the morphological traits provide a competitive advantage in the early stages. These factors, although some indirectly inferred, cannot be discarded, as they show the success and establishment of this species in new environments.

## Supporting Information

S1 Table Food items consumed (percentage volume)by *P*. *squamosissimus* developmental stages in Xambrê Lake, Upper Paraná River floodplain,Paraná State,Brazil. SL = standard Length;N = stomachs analyzed; Food items: Bacil = Bacillariophyta; Plant = Plant remains; Gastr = Gastropoda; Copep = Copepoda; Clado = Cladocera; Macro = *Macrobrachium amazonicum*; Chiron = Chironomidae; Ephem = Ephemeroptera; Trich = Trichoptera; Oaqin = Other aquatic insects; Psqua = *Plagioscion squamosissimus*; Ofish = Other fishes.
(DOCX)
