# Supplementary material for: Carnivory during Ontogeny of the Plagioscion squamosissimus: A Successful Non-Native Fish in a Lentic Environment of the Upper Paraná River Basin
Source: PLoS One. 2015 Nov 2;10(11):e0141651. doi: 10.1371/journal.pone.0141651 (PMC4629902; doi:10.1371/journal.pone.0141651)
Supplement: S1 Table — SL = standard Length;N = stomachs analyzed; Food items: Bacil = Bacillariophyta; Plant = Plant remains; Gastr = Gastropoda; Copep = Copepoda; Clado = Cladocera; Macro = Macrobrachium amazonicum; Chiron = Chironomidae; Ephem = Ephemeroptera; Trich = Trichoptera; Oaqin = Other aquatic insects; Psqua = Plagioscion squamosissimus; Ofish = Other fishes. (DOCX) [file pone.0141651.s001.docx]

**Supporting Information**

**Table S1. Food items consumed (percentage volume) by *P. squamosissimus* developmental stages in Xambrê Lake. Upper Paraná River floodplain. Paraná State. Brazil. SL= standard Length; N= stomachs analyzed; Food items: Bacil=Bacillariophyta; Plant= Plant remains; Gastr=Gastropoda; Copep= Copepoda; Clado=Cladocera; Macro=*Macrobrachium amazonicum*; Chiron=Chironomidae; Ephem= Ephemeroptera; Trich= Trichoptera; Oaqin =Other aquatic insects; Psqua=*Plagioscion squamosissimus*; Ofish=Other fishes.**

| Stages | SL (cm) | N | Food items | | | | | | | | | | | |
| --- | --- | --- | --- | --- | --- | --- | --- | --- | --- | --- | --- | --- | --- | --- |
|  |  |  | Bacil | Plant | Gastr | Copep | Clado | Macro | Chiron | Ephem | Trich | Oaqin | Psqua | Ofish |
| Pre-flexion | 0.3-0.39 | 3 | 33.3 |  |  | 66.7 |  |  |  |  |  |  |  |  |
| Pre-flexion | 0.4-0.49 | 14 | 21.2 |  |  | 21.2 | 57.6 |  |  |  |  |  |  |  |
| Flexion | 0.5-0.59 | 41 |  |  | 19.6 | 43.1 | 37.3 |  |  |  |  |  |  |  |
| Flexion | 0.6-0.69 | 32 |  |  | 9.8 | 31.7 | 58.5 |  |  |  |  |  |  |  |
| Flexion | 0.7-0.79 | 22 |  |  |  | 59.3 | 33.3 |  | 7.4 |  |  |  |  |  |
| Flexion | 0.8-0.89 | 17 |  |  |  | 57.8 | 42.2 |  |  |  |  |  |  |  |
| Post-flexion | 0.9-0.99 | 33 |  |  | 6.9 | 8 | 85.1 |  |  |  |  |  |  |  |
| Post-flexion | 1.0-1.09 | 22 |  |  | 7.4 | 13 | 79.6 |  |  |  |  |  |  |  |
| Post-flexion | 1.10-1.19 | 6 |  |  |  | 30 | 70 |  |  |  |  |  |  |  |
| Post-flexion | 1.20-1.29 | 11 |  |  |  | 59.5 | 40.5 |  |  |  |  |  |  |  |
| Post-flexion | 1.30-1.39 | 7 |  |  | 17.6 |  | 82.4 |  |  |  |  |  |  |  |
| Post-flexion | 1.40-1.49 | 6 |  |  |  | 57.1 | 42.9 |  |  |  |  |  |  |  |
| Post-flexion | 1.50-1.59 | 2 |  |  |  |  | 100 |  |  |  |  |  |  |  |
| Post-flexion | 1.60-1.69 | 1 |  |  |  |  | 100 |  |  |  |  |  |  |  |
| Post-flexion | 1.70-1.79 | 1 |  |  |  |  | 100 |  |  |  |  |  |  |  |
| Post-flexion | 1.80-1.89 | 1 |  |  |  |  | 100 |  |  |  |  |  |  |  |
| Juvenile | 2.00-2.99 | 8 |  |  |  | 34.5 | 17.2 | 48.3 |  |  |  |  |  |  |
| Juvenile | 3.00-3.99 | 28 |  | 0.5 |  |  |  | 55.1 | 20.4 |  |  | 15.1 | 1.2 | 7.7 |
| Juvenile | 4.00-4.99 | 35 |  | 0.3 |  |  |  | 57 | 10.6 |  |  | 5.5 | 10.3 | 16.3 |
| Juvenile | 5.00-5.99 | 24 |  | 0.5 |  |  |  | 46.9 | 8.3 |  |  | 5 | 26.1 | 13.2 |
| Juvenile | 6.00-6.99 | 10 |  |  |  |  |  | 59.9 | 0 |  |  | 3.2 | 28.6 | 8.4 |
| Juvenile | 7.00-7.99 | 12 |  | 0.8 |  |  |  | 51.9 | 4.4 | 2.2 |  | 0.9 | 33.1 | 6.6 |
| Juvenile | 8.00-8.99 | 3 |  |  |  |  |  | 86 | 0.3 |  |  |  | 13.6 |  |
| Juvenile | 9.00-9.99 | 1 |  |  |  |  |  | 100 |  |  |  |  |  |  |
| Juvenile | 11.0-11.9 | 1 |  | 2.4 |  |  |  | 93.5 | 4.1 |  |  |  |  |  |
| Juvenile | 12.0-12.9 | 6 |  | 0.3 |  |  |  | 40.7 | 2.5 | 3.4 | 0.3 |  | 52.8 |  |
| Adult | 15.0-15.9 | 1 |  |  |  |  |  | 100 |  |  |  |  |  |  |
| Adult | 16.0-16.9 | 1 |  | 9.1 |  |  |  | 69.7 | 6.1 |  |  | 15.2 |  |  |
| Adult | 17.0-17.9 | 2 |  |  |  |  |  | 100 |  |  |  |  |  |  |
| Adult | 18.0-18.9 | 2 |  | 0.8 |  |  |  | 88.7 |  |  |  | 10.5 |  |  |
| Adult | 19.0-19.9 | 1 |  |  |  |  |  | 100 |  |  |  |  |  |  |
| Adult | 20.0-20.9 | 1 |  |  |  |  |  | 100 |  |  |  |  |  |  |
| Adult | 21.0-21.9 | 1 |  |  |  |  |  | 99.5 |  |  |  | 0.5 |  |  |
| Adult | 22.0-22.9 | 4 |  | 5.8 |  |  |  | 79.3 |  |  |  |  | 14.9 |  |
| Adult | 23.0-23.9 | 5 |  | 1.3 |  |  |  | 85 |  |  |  | 0.2 |  | 13.5 |
| Adult | 24.0-24.9 | 4 |  | 1.6 |  |  |  | 84.3 |  |  |  | 5.4 | 6.7 | 2 |
| Adult | 25.0-25.9 | 4 |  |  |  |  |  | 74.6 |  |  |  |  |  | 25.4 |
| Adult | 27.0-27.9 | 2 |  |  |  |  |  | 78.4 |  |  |  |  |  | 21.6 |
| Adult | 28.0-28.9 | 2 |  | 23.8 |  |  |  | 76.2 |  |  |  |  |  |  |
| Adult | 34.0-34.9 | 1 |  |  |  |  |  | 0.8 |  |  |  |  |  | 99.2 |
